# Effect of Maternal Flavour Conditioning Combined with Organic and Inorganic Iron-Supplemented Creep Feed on Piglet Performance and Haemoglobin Status

**DOI:** 10.3390/ani14091263

**Published:** 2024-04-23

**Authors:** Ryan Kristen, Roslyn Bathgate, Greg M. Cronin, Evelyn Hall, Malcolm Possell, Cormac John O’Shea

**Affiliations:** 1 School of Life and Environmental Sciences, Faculty of Science, The University of Sydney, Sydney, NSW 2006, Australiamalcolm.possell@sydney.edu.au (M.P.); 2 Sydney School of Veterinary Sciences, Faculty of Science, The University of Sydney, Sydney, NSW 2006, Australia; roslyn.bathgate@sydney.edu.au (R.B.); evelyn.hall@sydney.edu.au (E.H.); 3 Faculty of Science and Health, Technological University of the Shannon, Athlone Campus, Dublin Road, Athlone, N37 HD68 Co Westmeath, Ireland

**Keywords:** iron deficiency anaemia, aniseed, sow, pig

## Abstract

**Simple Summary:**

Traditional treatment protocols for piglets suffering from iron deficiency anaemia are currently lacking from both cost, labour and welfare perspectives. Dietary iron supplements during the suckling phase are problematic due to variable habituation to creep feed consumption. Maternal flavour conditioning may resolve this variability. This study evaluated the role of anise flavoured, iron supplemented creep, with or without maternal anise conditioning on the iron status and growth performance of piglets. Anise flavoured, iron supplemented creep achieved comparable haemoglobin and body weight status to groups receiving an iron injection. These findings merit further evaluation of iron-fortified creep feed as a supplementary strategy for piglets.

**Abstract:**

Iron injections are vital but imperfect against iron deficiency anaemia (IDA). This experiment explored the effects on piglets of maternal flavour conditioning and the voluntary intake of anise flavoured, iron-supplemented creep feed compared with iron injections. The experiment was a 2 × 2 factorial arrangement: ±maternal exposure to dietary anise flavour and ±intramuscular injections of piglets. Twenty-three sows and their litters (242 piglets) were randomly allocated to one of four treatments (*n* = 5 or 6 per treatment): no flavour plus no injection (NF + NI); no flavour plus iron injection (NF + I); flavour plus no injection (F + NI); and flavour plus iron injection (F + I). All piglets could access anise flavoured, iron-supplemented creep feed (organic and inorganic forms) from D2 of birth. Sow feed intake and milk anethole concentration, piglet body weight (BW) and average daily gain (ADG), creep feed disappearance, piglet behavioural time budgets, and piglet blood glucose and haemoglobin concentrations were determined. Over the four-week study, the only significant differences found were that iron-injected piglets had reduced blood glucose (*p* = 0.036) on D14 and that maternal flavour provision increased the frequency of piglet creep feed interaction (*p* = 0.023) and decreased the frequency of suckling events (*p* = 0.009). In summary, maternal flavour conditioning reduced piglet creep feed neophobia without influencing consumption. The supplementation of creep feed with iron and anise flavour to piglets under the conditions of this trial was effective in preventing IDA, regardless of exposure to maternal flavouring conditioning.

## 1. Introduction

The development of IDA in suckling piglets is a well-known problem that is protected against shortly after birth through the provision of an intramuscular injection of an iron-containing compound. Deficiency is more pronounced in indoor systems whereas in outdoor farrowing systems piglets naturally ingest soil, thereby acquiring some oral iron. Iron deficiencies in piglets stem from a combination of limited iron reserves at birth [1], increased iron demand from rapid growth [2], limited external iron supply from maternal milk [3] and imperfect iron absorption due to the immaturity of the molecular mechanisms of iron metabolism [4]. Failure to provide iron supplementation incurs a greater susceptibility to piglet morbidity and growth hindrance due to IDA [5,6]. Typically, in the absence of iron supplementation, IDA occurs in piglets between 6 and 10 days post-partum [7] at average haemoglobin (Hb) concentrations below or equal to the anaemic threshold of 80 g/L [8]. Currently in indoor pork production, IDA is prevented by a single intramuscular neck injection of iron dextran (FeH_2_O_4_S; 1 mL; 200 mg/mL) within the first three days of life. However, in large-scale production settings, this practice is laborious, compromises immunity [9,10], facilitates disease transmission [11] and is potentially a welfare issue [12,13]. Previous research on oral supplementation via pastes and water supply demonstrated a limited capacity to ameliorate IDA without frequent dosing [14] and compromised overall piglet performance [15]. Additionally, increased handling times and the unpleasant metallic taste of high-dosage iron supplements may negatively impact piglet behaviour [16]. Furthermore, non-injected piglets subject to voluntary intake of oral iron supplements were all reported to exhibit anaemia within the first two weeks of life due to inadequate iron consumption [8,17,18]. In contrast, Maes et al. [11] demonstrated that an iron-rich diet (24% iron content) provided to non-injected piglets three times in specialised feeders reduced the variability of voluntary iron intake to successfully combat anaemia and even surpassed blood Hb concentrations when compared to iron-injected piglets. However, this protocol requires additional infrastructure per farrowing crate, adding to the cost of production. Piglets are born with insufficient glycogen stores beyond the first day of life and colostrum intake is primarily responsible for preventing hypoglycaemia [19,20]. Previous iron supplementation studies have not monitored piglet glucose trends throughout the nursery phase; however, this could be a valuable variable to capture as intestinal iron overload could lead to pathogen proliferation and, potentially, hypoglycaemia due to associated sepsis [21]. 

Maternal flavour conditioning is another stimulus utilised to increase neonatal feed intake where a flavour is fed to the dam during late gestation and throughout lactation. The prenatal exposure of piglets to flavours such as anise, which contains the aromatic compound anethole, via the maternal diet induced olfactory recognition and subsequently generated a postnatal preference [22,23,24]. This continuity of flavour recognition from late gestation to the post-partum lactation environment could act as a sensory link, thereby reducing piglet neophobia via chemosensory conditioning [25]. Additionally, anise flavourings are readily accepted by pigs [26] and can also act as a masking agent, covering unpleasant, bitter-tasting inorganic iron supplements [27]. Combinations of inorganic and organic iron supplementation have been previously used in studies to provide a balanced delivery of iron supplementation [28]. It was hypothesised that maternal flavour conditioning with anise would stimulate creep feed consumption and thereby prevent IDA in piglets not receiving iron injections. The aim of this study was to determine the effect of maternal diet supplementation with an anise flavour on the consumption of iron-supplemented, anise-flavoured creep and piglet Hb and growth status.

## 2. Materials and Methods

### 2.1. Animal Ethics and Experimental Design

**Figure 1:**
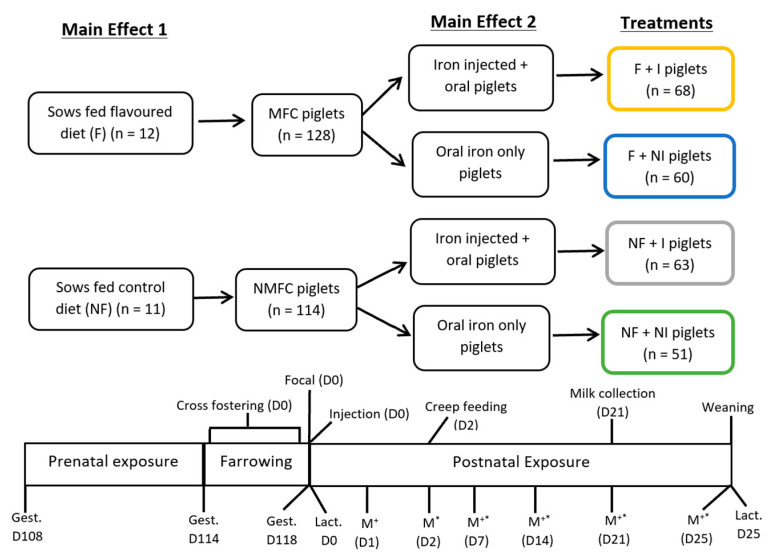
The experimental design and the times of the different treatments and procedures. MFC piglets = pigs from sows that received flavour feed during gestation and lactation; NMFC piglets = pigs from sows that received non-flavoured feed during gestation and lactation; NF + NI = piglets not exposed to maternal flavour conditioning and not injected with iron; NF + I = piglets not exposed to maternal flavour conditioning and injected with iron; F + NI = piglets exposed to maternal flavour condition and not injected with iron; F + I = piglets exposed to maternal flavour conditioning and injected with iron; Gest. = gestation; Focal = selection of focal piglets for blood collections; Inj = administration of iron injection; Lact. = lactation; M^+^ = performance and blood measurements; M* = behavioural measurements; M^+^* = performance, blood and behavioural measurements.

The University of Sydney Animal Ethics Committee approved this experiment (2017/project 1256), which was conducted at the University of Sydney piggery in a 2 × 2 factorial arrangement, with experimental treatments assigned randomly across three successive farrowing batches. The main effects evaluated were (1) sow feed anise flavour added (F) versus no added flavour (NF) and (2) iron injection (I) of the litter versus no iron injection (NI). Hence, twenty-three large white-landrace sows and their litters (242 piglets) were randomly allocated to one of four treatments (*n* = 5 or 6 per treatment): (1) no sow flavour plus no injection (NF + NI); (2) no flavour plus iron injection (NF + I); (3) flavour plus no injection (F + NI); and (4) flavour plus iron injection (F + I). A control group receiving no iron whatsoever was not considered in this study due to animal ethics considerations of subjecting piglets to IDA. A previous pilot study conducted by this research group confirmed the development of IDA in intensively housed piglets in the absence of any iron supplementation. Sows were housed individually in farrowing crates (1.6 × 2.2 m) one week before expected farrowing (day 108 of gestation) and randomly assigned to a standard gestation/lactation control diet (2.8 kg/day once per day until farrowing and then gradually to appetite) with no flavour (NF; *n* = 11) or an identical ration to which anise flavour (Aniseed extract; AFIS, Brookvale, NSW, Australia) was added (F; *n* = 12) until piglets were weaned (25 days of age). The feed intake of both control and flavour-fed sows was determined daily from day 108 of gestation until day 25 of lactation by weighing residual feed each day prior to the provision of fresh feed and subtracting the residual feed weight from the original feed weight provided. All sows farrowed within a four-day range (gestational days 114–118 producing 262 piglets in total before assignment to treatments) and litters were randomly assigned to iron treatments of injectable or oral iron supplementation (Figure 1). Litter sizes at farrowing ranged from 6 to 18 piglets and were balanced through cross-fostering within treatment shortly after birth, resulting in 11.4 piglets per litter with a ratio of male to female of 1:1. Piglets that were unable to be cross-fostered into the same treatment group were excluded from the study (*n* = 20). Between 24 and 36 h postpartum, piglets were weighed, ear notched for identification, tail-docked (approximately one-third of tail) and teeth-clipped in line with the farm husbandry practises. Also, within the 24–36 h postpartum period, piglets (*n* = 128) were assigned to the injectable iron supplementation group (Feron 200 + B12; Bayer) and received a 1 mL intramuscular iron injection (200 mg/mL iron dextran and 40 μg/L cyanocobalamin). The remaining piglets (*n* = 114) were then assigned solely to the oral iron supplementation protocol with no intramuscular injection of iron dextran. During lactation, litters were housed with their parental sow (unless cross-fostered) in conventional farrowing crates in an environmentally controlled room containing eight crates with fully slatted, tri-bar metal flooring. Rubber matting provided an area of solid flooring (0.5 × 1.8 m) in the heated creep area. A portion (0.5 kg) of anise-flavoured and iron-supplemented creep feed was provided daily at 08:00 h in a shallow tray (330 × 230 × 20 mm) fixed to the rubber matting from day 2 for all litters, along with ad libitum water access via a piglet nipple drinker. The utilisation of creep feed was determined daily from day 2 until weaning (day 25) by weighing residual creep feed in the trays each day prior to the provision of fresh feed and subtracting the residual weight from the amount provided per day. The body weight of piglets was measured at birth and on days 7, 14, 21 and 25 to calculate the average daily gain (ADG).

### 2.2. Flavour Exposure and Creep Composition

The experimental flavour chosen was anise, which contains the aromatic compound anethole (C_10_H_12_O). All sows in the flavour treatment group (F) received commercial sow feed mixed daily with food-grade anise flavour (Aniseed extract; AFIS, Brookvale, NSW, Australia), dosed at 1 mL of flavour per kg of feed (53 ± 1.2 g/L of anethole in solution). A protocol was followed to prevent exposure of the control sows to the anisic flavour that consisted of feeding the control sows before the flavour-fed sows, wearing gloves, distributing flavoured and control feed via different scoops, and mixing flavour with commercial feed outside the facility. From day 2 until weaning (25 days of age), all piglets received commercial creep feed (Blueprint 900; Alltech, Roseworthy, SA, Australia) containing 15.9 MJ/kg, 23% min CP and added iron of 55 mg/kg (Bioplex 15%; Alltech, Roseworthy, SA, Australia), to which supplemental iron was added. The concentration of supplemental iron was determined by aiming to achieve 200 mg of dietary iron being retained per piglet in the early stages of the suckling period, hence analogous to the 200 mg of iron delivered intramuscularly shortly after birth. An average daily creep intake of 4 g per piglet per day and an approximate retention rate of 50% for orally ingested iron were assumed. All creep feed portions were prepared individually and made up in batches of 500 g, including a top dressing of 16 g of organic iron chelate (Bioplex 15%; Alltech, Roseworthy, SA, Australia) and 32 g of inorganic iron sulphate (Ferrous Sulphate mono 30%; Alltech, Roseworthy, SA, Australia). The total iron content of the creep was estimated to be 25 g/kg, giving an approximate dietary intake of 100 mg per piglet per day. Anise was also added to all creep feed with the same anise flavour product (Aniseed extract; AFIS, Brookvale, NSW, Australia), dosed at 2 mL of flavour per kg of creep feed (53 ± 1.2 g/L) of anethole in solution.

### 2.3. Collection of Blood for Physiological Measurements

Once farrowing was completed, four focal piglets were selected within each litter. The focal piglets selected were two males and two females closest to the average body weight for their respective litter. These focal piglets were used for the collection of blood to measure Hb and glucose concentrations. Blood was sampled aseptically (70% ethanol) from lanced ear capillaries at birth before any iron administration. Subsequent blood samples were collected from lanced ear capillaries throughout the study on days 7, 14, 21 and 25 from the same four piglets. A few drops of capillary blood were aspirated by capillary force into a disposable microcuvette. Haemoglobin concentrations (g/L) and glucose concentrations (mmol/L) in these samples were determined by means of a photometer, and specific microcuvettes were loaded into their respective measurement device (HemoCue^®^: Hb201 + and Glucose 201 + System, Angelholm, Sweden). The photometer was calibrated during manufacture using the haemoglobin cyanide method for haemoglobin concentration measurement and the ID gas-chromatography mass-spectrometry (GC-MS) method for glucose concentration measurement. The photometers were also tested for quality control via their respective control standards after each farrowing batch.

### 2.4. Behavioural Measurements

In each litter, piglets were individually numbered on the back using a felt-tipped pen. Piglets were thus marked on the day prior to scheduled behavioural measurements. Identifying individual piglets enabled the quantification of total creep feed interactions by specific piglets within litters. Behaviours were scored live using an instantaneous scan sampling method at 3 min intervals for 2 h per day (40 observations/d) on days 2, 7, 14, 21 and 25. The information recorded included postures at the creep tray, interactions with the creep tray, activity at the sow’s udder and the number of suckling bouts (Table 1). Observations commenced at 08:00 h, 1 h after sow feeding, giving sows ample time to consume their feed, so that sow standing would not confound piglet behavioural data. The observers were blind to the treatments of the different crates and trained before the experiment and a high inter-observer reliability was achieved within and across batches.

### 2.5. Determination of Anethole in Food-Grade Flavouring Agent

The aniseed concentration in the food-grade anise flavouring agent was determined by GC-MS. One μL of the flavour substance was injected using a multipurpose sampler (MPS; Gerstel, Mülheim an der Ruhr, Germany) into a programmed temperature vaporisation (PTV) inlet (CIS-4; Gerstel) installed in an Agilent 7890GC (Agilent Technologies Pty Ltd., Mulgrave, VIC, Australia), which was used in solvent mode. The injection syringe was rinsed with ethanol (Sigma-Aldrich; Sydney, NSW, Australia) between sample injections. The PTV inlet containing a glass liner filled with Tenax TA was held at 20 °C during the injection, using liquid CO_2_ (BOC Ltd., North Ryde, NSW, Australia) as the cryogen. After the injection was complete, the CIS-4 was heated at 12 °C s^−1^ to 300 °C and held at that temperature for 5 min while the flavours were injected into the GC at a 100:1 split ratio. Flavours were separated on an HP-5 ms capillary column (30 m × 0.25 mm, 0.25 μm film thickness; Agilent), which was connected to a two-way splitter with makeup gas (Agilent). A restrictor column of deactivated fused silica (1.44 m × 0.18 mm; Agilent), connected to the outlet of the splitter, transferred the compounds to a mass-selective detector (Model 5975C; Agilent). Ultra-high-purity helium was used as the carrier gas (flow rate through the HP-5 ms column was 2.3 mL min–1 and 4 mL min-1 through the restrictor column). The initial oven temperature of the GC was 35 °C, which was held for 5 min and then heated at a rate of 4 °C min^−1^ to 160 °C, then at 20 °C min^−1^ to 300 °C and held isothermal for 5 min. The temperature of the GC-MS interface was 280 °C, the MS ion source was 230 °C and the quadrupole was 150 °C. The detector, in electron impact mode (70 eV), scanned the range of 35–450 *m*/*z*. Operation of the GC-MS was controlled by Agilent Chemstation (version E.02.01.117) and the TDU was controlled by Maestro (version 1.4.36.16; Gerstel). The quantification of anethole was performed by selected ion monitoring of the dominant three ion fragments of anethole (148, 147 and 117 *m*/*z*). The calibration curve of anethole (dissolved in ethanol; Sigma-Aldrich; Sydney, NSW, Australia) was linear over the calibration range (0–7%), with an R^2^ of 0.995 and a detection limit of 0.02%.

### 2.6. Collection and Quantification of Anethole in Sow Milk

During peak lactation (day 21 post-parturition) in the second replicate, a total of 15 milk samples were collected from four flavour-fed and four control sows over 3 h post-feed ingestion via hand milking across multiple teats. Previous research has demonstrated the dietary transfer of anethole into human breast milk, with the highest anethole concentration occurring 2 h post-ingestion (Hausner et al., 2008 [29]). However, two additional milk samples were collected from one flavour-fed sow 4 h post-ingestion. All hand-collected samples were immediately frozen (−20 °C) and stored until laboratory analysis. Analysis of the concentration of anethole present within the sow milk was determined using a Gerstel Thermal Desorption Unit (TDU; Gerstel, Mülheim an der Ruhr, Germany). Fifty microlitres of milk from each sample was pipetted into individual microvials that were placed into separate glass thermal desorption liners. These liners were inserted into the TDU for analysis. Upon insertion into the TDU, the samples were purged with ultra-high-purity helium (BOC Ltd., North Ryde, NSW, Australia) at 30 °C for 1 min to eliminate air from the sample and inlet. Samples were then heated by the TDU at 12 °C s^−1^ to 70 °C with a helium flow of 60mL min-1. Thermal desorption (TD) products were carried by the helium through to a programmed temperature vaporisation (PTV) inlet (CIS-4; Gerstel) as described above. The PTV, GC and MS programs were the same as for the aniseed flavour analysis, except the injection from the CIS-4 to the GC column was performed in a splitless manner.

### 2.7. Statistical Analysis

All statistical analyses were conducted with GenStat (ver 18.2, VSN International Ltd., Hemel Hempstead, UK) and a *p*-value of <0.05 was considered significant. Performance, blood and behavioural data were analysed using linear mixed-effects modelling. The fixed effects considered in each model were treatment group, day, and their interaction, with a random effect of batch nested within crate and sow number. Body weight measurements were developed into ADG data to standardise each litter’s basal body weight measurements. Focal piglet data were used to estimate blood haemoglobin and glucose concentrations for their respective litter, which were then developed into a rate of change dataset to standardise litters’ basal haemoglobin and glucose measurements. Behavioural observations were expressed as success rates for each studied behaviour (Table 1), which were then divided by litter size and multiplied by the total number of observations. This value provided a proportion of time that each piglet spent performing each behaviour, which was then analysed by linear mixed modelling as above. Data normality was assessed using the Anderson–Darling test, with data not meeting requirements for normality being log-transformed accordingly. Predicted means from the modelling were back-transformed as required and presented as mean ± standard error unless stated otherwise. Descriptive analysis was used to identify the presence and concentration of anethole within sow milk.

## 3. Results

### 3.1. Performance Measurements

**Figure 2:**
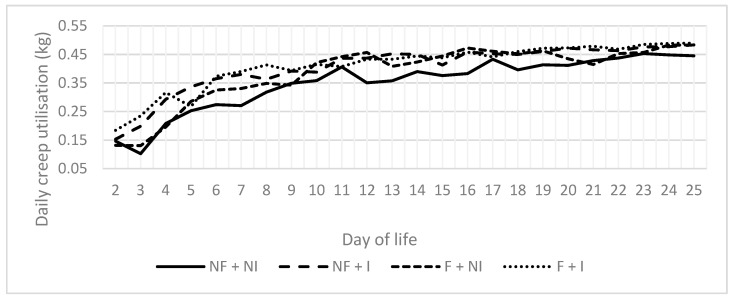
Effects of maternal flavour conditioning and iron administration treatments on averaged daily litter creep feed utilisation from all litters assigned to their corresponding treatment groups within the three replicates. No flavour exposure and non-injectable iron (NF + NI). No flavour exposure and injectable iron (NF + I). Flavour exposure and non-injectable iron (F + NI). Flavour exposure and injectable iron (F + I).

**Figure 3:**
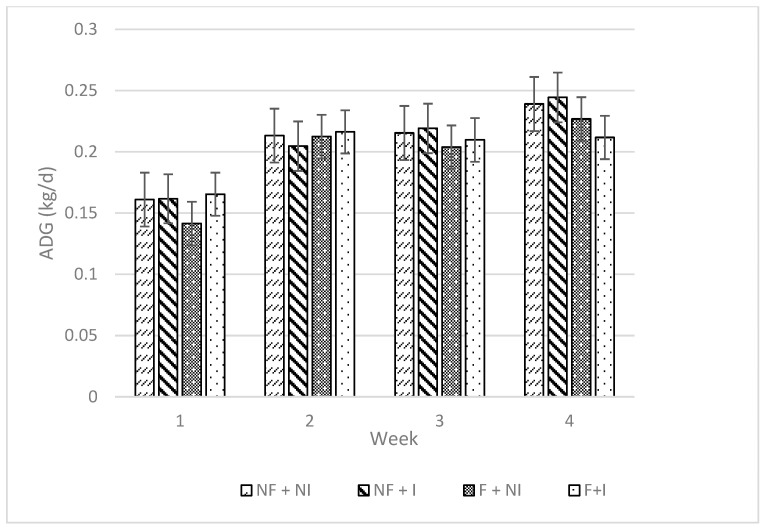
Effects of maternal flavour conditioning and iron administration treatments on piglet ADG over the study. No flavour exposure and non-injectable iron (NF + NI). No flavour exposure and injectable iron (NF + I). Flavour exposure and non-injectable iron (F + NI). Flavour exposure and injectable iron (F + I). Week 1 (days 0–7), Week 2 (days 7–14), Week 3 (days 14–21) and Week 4 (days 21–25).

**Figure 4:**
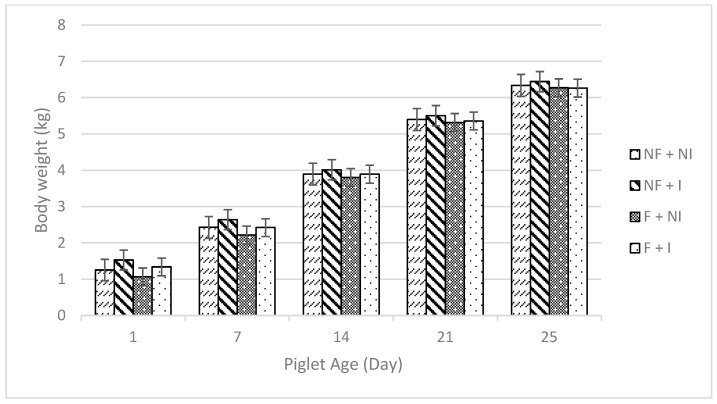
Effects of maternal flavour conditioning and iron administration treatments on piglet body weight over time until weaning (day 25). No flavour exposure and non-injectable iron (NF + NI). No flavour exposure and injectable iron (NF + I). Flavour exposure and non-injectable iron (F + NI). Flavour exposure and injectable iron (F + I).

Maternal flavour conditioning, iron supplementation, and their interaction had no significant effects on all performance measures including sow feed intake (Table 2), creep feed utilisation (Table 2; Figure 2) and average daily gain (Table 2; Figure 3). All treatments demonstrated typical weight gain (Figure 4) and, due to a high correlation between body weight and ADG (0.7939), results are only presented for ADG data.

### 3.2. Blood Parameter Measurements

#### 3.2.1. Haemoglobin (Hb) Concentrations

**Figure 5:**
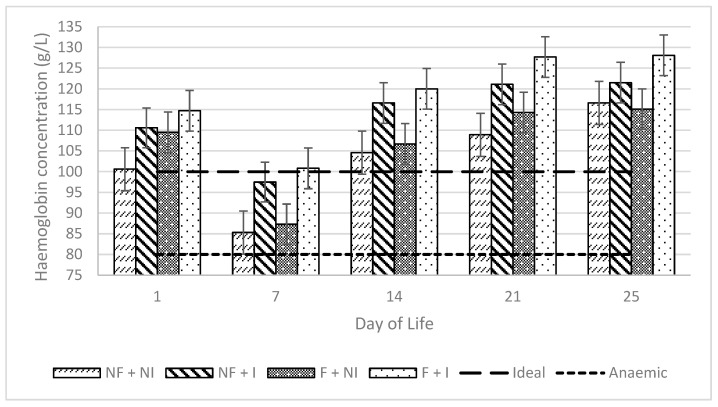
Effects of maternal flavour conditioning and iron administration treatments on piglet haemoglobin concentrations over time. No flavour exposure and non-injectable iron (NF + NI). No flavour exposure and injectable iron (NF + I). Flavour exposure and non-injectable iron (F + NI). Flavour exposure and injectable iron (F + I). Ideal is the recommended haemoglobin concentration for healthy piglets and IDA is seen once haemoglobin concentrations fall below 80 g/L of blood [8].

**Figure 6:**
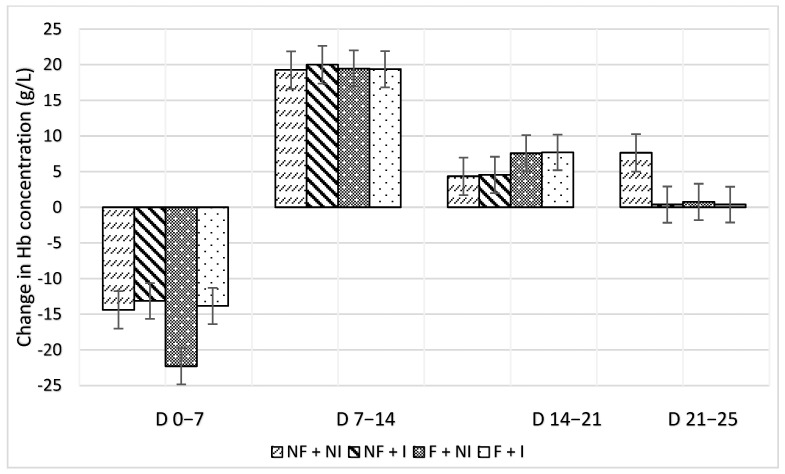
Effects of maternal flavour conditioning and iron administration treatments on change in piglet haemoglobin concentrations over time. No flavour exposure and non-injectable iron (NF + NI). No flavour exposure and injectable iron (NF + I). Flavour exposure and non-injectable iron (F + NI). Flavour exposure and injectable iron (F + I). Week 1 (days 0–7), Week 2 (days 7–14), Week 3 (days 14–21) and Week 4 (days 21–25).

Maternal flavour conditioning, iron supplementation and their interaction had no significant effects on average or weekly changes in haemoglobin concentrations (Table 2). All treatments prevented haemoglobin concentrations from falling below 80 g/L of blood, therefore averting the presence of clinical IDA within litters (Figure 5). Additionally, Hb was found not to be significantly correlated with either ADG (0.21) or blood glucose concentrations (−0.31). Over-time change in Hb was similar between iron administration treatments (Figure 6; Table 2).

#### 3.2.2. Glucose Concentrations

**Figure 7:**
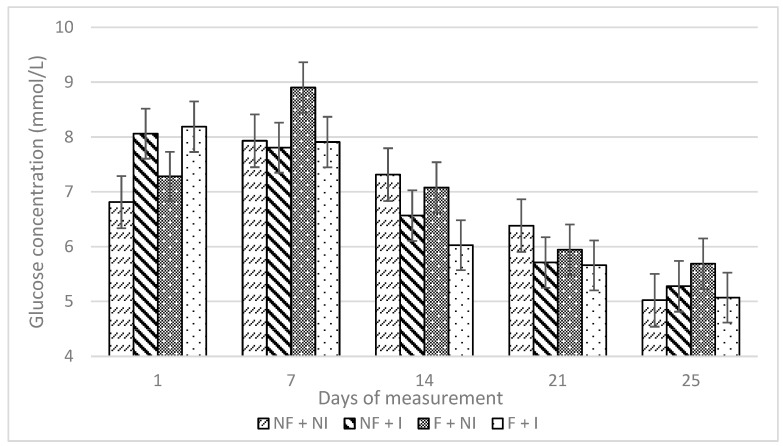
Effects of maternal flavour conditioning and iron administration treatments on piglet blood glucose concentrations over time. No flavour exposure and non-injectable iron (NF + NI). No flavour exposure and injectable iron (NF + I). Flavour exposure and non-injectable iron (F + NI). Flavour exposure and injectable iron (F + I).

**Figure 8:**
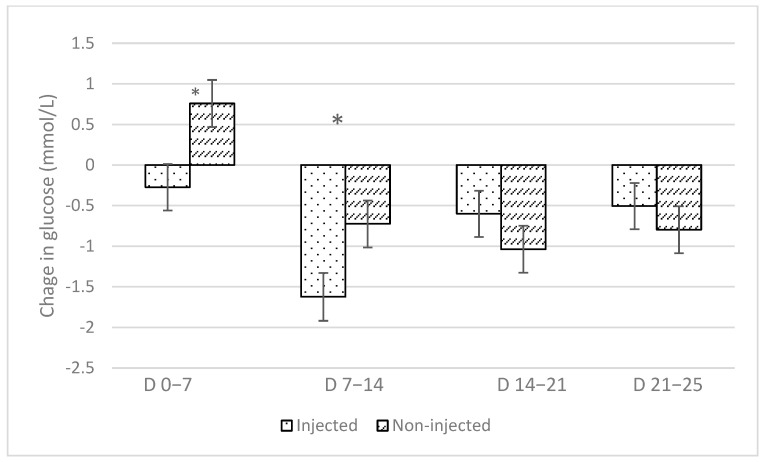
Effect of iron supplementation protocol on change in piglet blood glucose concentrations over time. Week 1 (days 0–7), Week 2 (days 7–14), Week 3 (days 14–21) and Week 4 (days 21–25). * indicates a significant difference (*p* < 0.05).

Flavour and flavour interaction with iron supplementation had no significant effect on average or weekly changes in glucose concentrations (Table 2). All treatments demonstrated relatively constant blood glucose concentrations (Figure 7). Iron supplementation had no effect on average glucose concentrations but significantly affected weekly changes in glucose concentrations (Table 2) as non-injected piglets had a positive change in glucose concentrations in the first week (non-injected, 0.758 mmol/L ± 0.290 and injected, −0.274 mmol/L ± 0.285) and a reduced negative change in glucose concentrations in the second week (non-injected, −0.725 mmol/L ± 0.290 and injected, −1.624 mmol/L ± 0.295) compared with the injected piglets (Figure 8).

### 3.3. Behavioural Observations 

**Figure 9:**
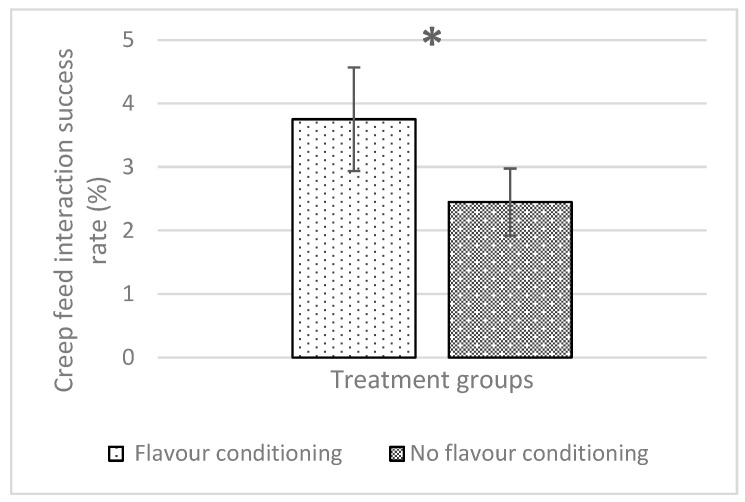
Effect of maternal flavour conditioning on the overall success of piglet–creep interactions. * indicates significant difference (*p* < 0.05).

**Figure 10:**
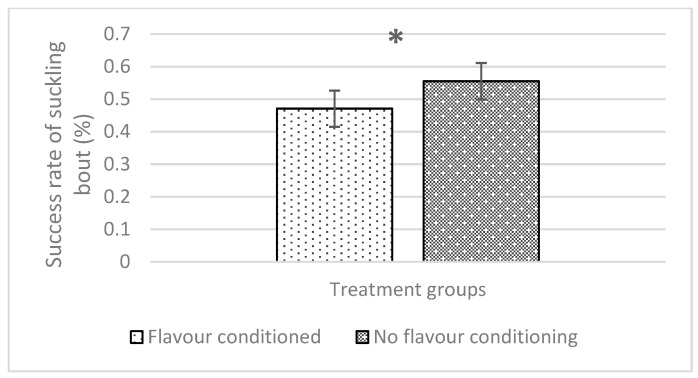
Effect of maternal flavour conditioning on the overall success suckling bouts with milk letdown. * indicates significant difference (*p* < 0.05).

All interactions between the main effect treatment combinations (maternal flavour conditioning, iron administration method and week) were not significant for any piglet behaviour (Table 3). The iron administration method had no effect on any behaviour recorded in this experiment. However, maternal flavour conditioning had a significant effect on creep feed interactions and suckling bout behaviours (*p* = 0.023 and *p* = 0.009, respectively). Therefore, piglets subject to flavour conditioning spent more time interacting with creep feed during behavioural observations (flavour, 3.751% ± 0.814 and no flavour, 2.448% ± 0.529; Figure 9). Additionally, piglets subject to flavour conditioning spent less time suckling milk from their sow fed the anise-flavoured diet (flavour, 0.471% ± 0.056 and no flavour, 0.555% ± 0.056; Figure 10).

### 3.4. Anethole Concentrations in Flavouring Agent and Sow Milk

Large amounts of anethole were detected in the food-grade anise flavouring (Aniseed extract; AFIS). The standard was found to contain 53 ± 1.2 g/L of anethole, as well as trace amounts of terpenes such as cymene, limonene and myrcene. No detection of anethole was found in the milk of all control sows and three flavour-fed sows within the 3 h sampling period post-feed ingestion. However, an average anethole concentration of 50 ppm was identified within the milk of one flavour-fed sow at 4 h post-feed ingestion. 

## 4. Discussion

This pilot study demonstrated that supplying piglets with anise-flavoured creep feed containing organic and inorganic iron showed potential as a means of achieving iron supplementation in non-injected pigs. Without supplementation, IDA would be considered the most prevalent micronutrient deficiency disorder within intensive pig production systems [5]. Current iron supplementation husbandry practises have several potentially adverse repercussions such as iron toxicity, inducing oxidative stress [5], secondary infections predisposing piglets to arthritis [10], elevated production costs associated with individual supplementation protocols [30] and compromised welfare linked with needlestick injuries and poor hygiene [11]. Previous attempts at voluntary oral iron supplementation have demonstrated disappointing piglet responses, resulting in insufficient iron intake to combat IDA [8,18]. In attempting to overcome this by the addition of flavour masking agents, this study demonstrated that flavour-conditioned piglets had increased interactions with anise-flavoured creep feed but did not increase their creep consumption. Despite this, providing non-injected piglets with an anise-flavoured, iron-supplemented creep diet resulted in growth patterns and blood haemoglobin concentrations similar to those observed with intramuscular iron dextran injection. 

General consensus classifies a piglet as anaemic once the reduced haemoglobin concentration depresses ADG or the piglet expresses clinical signs such as pale skin. This corresponds with haemoglobin concentrations of less than 80 g/L [30]. Piglets with haemoglobin concentrations of 100 g/L are considered within normal range and those with values of 80–99 g/L are considered to have sub-clinical anaemia [30]. Our findings are congruent with the literature as the lowest haemoglobin concentration in the NI + NF treatment declined to 85 g/L during the first week of life. This treatment group did not display any signs of clinical anaemia or depression in ADG and all piglets, regardless of the treatment group, achieved similar growth of 150 g per day during the first week of life, aligning with Australian performance standards [31].

The general decline in haemoglobin concentrations across treatment groups in the first week after parturition corroborates with literature stating that the limited iron in sow milk, plasma expansion and rapid growth all contribute to this negative change in haemoglobin concentrations in piglets [8,15]. All piglets had access to iron-supplemented creep feed, while haemoglobin concentrations and ADG of piglets receiving no iron injections were comparable to piglets receiving standard iron injections shortly after birth. These results align with previous research conducted by Maes et al. [11], who first demonstrated that the combined effects of organic and inorganic supplements in creep feed provisions were able to combat anaemia via voluntary intake with specially designed feeders. 

Previous research exploring maternally derived flavour conditioning showed that piglets displayed flavour recognition after less than 10 days of exposure during the last trimester of gestation [22,32]. In that study, flavour-conditioned piglets congregated in flavoured areas, with fewer vocalisations and escape attempts than control piglets (non-flavour-conditioned) when faced with a stressful and unfamiliar environment. The behavioural results in the current study support this concept of maternal flavour conditioning as flavour exposure during late gestation and throughout lactation almost doubled the proportion of time each piglet spent interacting with the flavoured creep feed. However, this increase in creep feed interactions did not result in increased creep consumption. Further work in this area should investigate ways to encourage intake of the iron-supplemented creep feed, perhaps in the form of different pellet sizes or consistencies.

Pigs are known to have high taste acuity [33], so the inclusion of aversive flavoured iron supplements could reduce creep palatability and impinge on piglet voluntary intake of creep feed. This adverse response of piglets to oral iron supplementation has been previously reported by Svodoba and Drabek [18] as they demonstrated insufficient voluntary intake of inorganic iron supplements that resulted in numerous cases of piglet IDA. Anise has been reported to act as a masking agent [27], which could have suppressed the aversive taste associated with oral iron supplements [34] and standardised creep feed intake across all treatments. The disappearance of creep feed throughout lactation in this study corroborates with the comparable studies [35] as piglets made minimal use of creep feed in the early days of life (100–150 g/day), but this gradually rose to 400 g/day around weaning (day 25 of life). Therefore, the inclusion of anise flavouring, whether maternal exposure to anise was present or not, may have increased feed palatability [26], leading to the sufficient intake of oral iron, so outcomes were comparable to piglets receiving injected iron. Further research exploring the masking effect of anise flavouring on iron-supplemented creep feed in the absence of maternal flavour conditioning is required to confirm any palatability improvements in supplemented creep. Further adding to the evidence for placental and lactational exposure leading to flavour conditioning, piglets whose dam had pre- and post-gestational exposure to anise spent less time at the udder during milk letdowns, dedicating more time towards playing or foraging in creep feed. This result is in accordance with the literature, where the presence of the flavour in feed increased the frequency of play and foraging behaviours at weaning in piglets that were perinatally exposed to the same flavour [24]. Despite not impacting the body weight status of those piglets, a reduction in udder time is not a desirable outcome. Due to the limited replication and narrow observation intervals in the current study, the full extent of this trend may not have been apparent. To account for this, further replication will need to be undertaken along with continuous daily video monitoring to accurately assess piglet behaviours. 

Interestingly, within the first two weeks after parturition, the oral iron administration protocol had a positive effect on piglet blood glucose concentrations. While speculative, these changes in glucose concentrations may be attributed to the enhanced use of creep feed in the absence of injectable iron protocols. The pain associated with injectable iron supplementation might have altered feeding behaviours, potentially suppressing early feed intake and subsequently leading to decreased blood glucose concentrations within the first 14 days of life. Increased glucose concentrations through increased feed consumption within the first few weeks of life can lead to improved piglet survival and immunity, especially via colostrum intake [20]. Future research to investigate this change in glucose concentrations could include the addition of titanium dioxide to creep feed to elucidate the primary source of energy for piglets during the first two weeks of lactation.

Anethole is a phytogenic feed compound mainly extracted from aniseed (*Pimpinella anisum*) and fennel (*Foeniculum vulgare*) and has been routinely used in flavour conditioning studies in species such as rodents [36], pigs [24], dogs [37] and lambs [38]. Few of the studies on swine have confirmed the concept of volatile aromatic compounds transferring from the maternal diet to either sow milk [39] or amniotic fluid [40]. In the current study, only one sow was shown to have transmitted anethole from the maternal diet to milk and this transmission was minimal, as seen previously [39]. A study in the differential transfer of dietary compounds into human breast milk has confirmed that flavour type, individual differences and food consumption are important regulating factors of transmission quantity and speed [29]. This may explain the failure of anethole detection in milk from the other flavour-fed sows in the study as the duration of the sampling period may have been inadequate. Further research is required to characterise this phenomenon due to the unrepeatable confirmation of anethole transmission from the sow’s diet to her milk. Using simple on-farm devices (Hemocue) to monitor capillary blood haemoglobin concentrations in real time can only provide an outline of the overall picture of IDA [30]. A more invasive but holistic approach is to analyse piglet blood for packed cell volume and erythrocyte indices via jugular collections. Furthermore, serum examination from jugular collections enables the diagnosis of subclinical IDA, identifying either pre-latent or latent iron deficiency via biochemical markers of iron metabolism such as serum total iron binding capacity, transferrin, transferrin saturation ferritin, serum soluble transferrin receptor and, more recently, plasma hepcidin-25 [4]. Hepcidin is a peptide hormone synthesized by hepatocytes in the liver to negatively regulate dietary iron absorption in enterocytes based on transferrin saturation by binding and subsequently degrading ferroportin to prevent intestinal iron absorption [41]. Starzyński et al. [4] demonstrated that traditional iron dextran injections can rectify IDA in piglets but are accompanied by the risk of excessive hepcidin expression, impairing the absorption of dietary iron. An area of future interest would be assessing plasma hepcidin-25 in piglets given the iron dietary supplement used here as a multiple low-dosage supplementation may yield similar results to the modified protocol of split iron dextran supplementation developed by Starzyński et al. [4]. The importance of this comparison based on hepcidin expression is that a second iron dextran injection is labour-consuming and further imposes on welfare, so it is less appealing to the industry in comparison with the voluntary oral iron supplementation protocol developed in this study. 

Maes et al. [11] reported that intramuscular injections of iron dextran take up to 30 s longer per litter than oral iron administration, which is of economic importance in large-scale production settings because of labour considerations. That study demonstrated similar haemoglobin concentrations and growth performance parameters in piglets dosed with voluntary oral iron when compared with iron dextran injections. However, the piglets were encouraged to intake oral iron via the installation of specialised creep feeders that required ongoing cleaning. This negated any labour savings from the removal of the iron injection. In the present study, comparable haemoglobin concentrations were achieved without the need for additional infrastructure, saving on initial installation costs and ongoing maintenance. Additionally, the application of this oral iron top dressing pre- or post-pelleting improves biosecurity as it can be applied without stepping into pens, so pathogen transmission between litters is minimised.

The results presented here demonstrate the potential for replacing the industry-standard iron dextran injections with oral iron supplementation, subject to validation with follow-up experimental and field studies. Additionally, confirmation of the cost-effectiveness of this protocol and the lifetime growth parameters of the pigs should be investigated before commercial uptake is warranted. 

## 5. Conclusions

These results demonstrated that voluntary intake of iron provided daily through creep feed creates an appealing alternative to traditional intramuscular iron dextran injections. There were equal haemoglobin concentrations and ADG between litters of both control (injected) and non-injected treatments. Finally, maternal flavour conditioning increased creep feed interactions and reduced piglet neophobia but reduced suckling bouts and failed to increase creep feed consumption of anise-conditioned piglets. The ability to prevent IDA without iron dextran injections is promising for the future industry adoption of this protocol in routine piglet care.

## Figures and Tables

**Table 1:** Behaviour and posture categories scored during live farrowing crate observations.

Behaviour	Posture	Definition
Interaction with creep	Stand	Piglet standing with at least one front foot in the tray
	Lie	Piglet lying with at least 50 per cent of their body in the tray
	**Udder activity**	
Maternal interactions	Active	Piglet actively suckling teat (no milk letdown) or nosing udder of sow
	Inactive	Piglet present at udder but no interaction with udder (asleep or walking past and under sow)
	Suckle	Piglet received milk letdown during suckling episode at the teat

**Table 2:** *p*-values from linear mixed-effects modelling of performance and blood parameter data based on the effects of maternal flavour conditioning, iron administration protocol and their interactions. * indicates a significant difference (*p* < 0.05).

		Flavour	Iron	Interaction
Sow intake	Daily	0.088	0.932	0.7
	Total	0.52	0.132	0.389
Creep feed	Daily	0.976	0.136	0.785
	Total	0.688	0.402	0.747
ADG	Overall	0.633	0.945	0.924
Haemoglobin	Average	0.652	0.859	0.321
	Weekly delta	0.146	0.126	0.531
Glucose	Average	0.882	0.132	0.846
	Weekly delta	0.564	0.0036 *	0.478

**Table 3:** *p*-values from linear mixed-effects modelling of behavioural data based on the effects of maternal flavour conditioning, iron administration protocol and their interactions. * indicates significant difference between treatment groups (*p* < 0.05).

Behaviour	Flavour	Iron	Interaction
Creep interaction	0.023 *	0.417	0.756
Creep stand	0.141	0.539	0.837
Creep lie	0.410	0.459	0.932
Udder active	0.711	0.103	0.746
Udder inactive	0.998	0.139	0.677
Suckling bout	0.009 *	0.383	0.241

## Data Availability

The raw data supporting the conclusions of this article will be made available by the authors upon request.
